# Development of Multisensory Reweighting Is Impaired for Quiet Stance Control in Children with Developmental Coordination Disorder (DCD)

**DOI:** 10.1371/journal.pone.0040932

**Published:** 2012-07-18

**Authors:** Woei-Nan Bair, Tim Kiemel, John J. Jeka, Jane E. Clark

**Affiliations:** 1 Department of Physical Therapy and Rehabilitation Science, University of Maryland, Baltimore, Baltimore, Maryland, United States of America; 2 Department of Kinesiology, University of Maryland, College Park, College Park, Maryland, United States of America; 3 Program in Neuroscience and Cognitive Sciences, University of Maryland, College Park, College Park, Maryland, United States of America; The University of Western Ontario, Canada

## Abstract

**Background:**

Developmental Coordination Disorder (DCD) is a leading movement disorder in children that commonly involves poor postural control. Multisensory integration deficit, especially the inability to adaptively reweight to changing sensory conditions, has been proposed as a possible mechanism but with insufficient characterization. Empirical quantification of reweighting significantly advances our understanding of its developmental onset and improves the characterization of its difference in children with DCD compared to their typically developing (TD) peers.

**Methodology/Principal Findings:**

Twenty children with DCD (6.6 to 11.8 years) were tested with a protocol in which visual scene and touch bar simultaneously oscillateded medio-laterally at different frequencies and various amplitudes. Their data were compared to data on TD children (4.2 to 10.8 years) from a previous study. Gains and phases were calculated for medio-lateral responses of the head and center of mass to both sensory stimuli. Gains and phases were simultaneously fitted by linear functions of age for each amplitude condition, segment, modality and group. Fitted gains and phases at two comparison ages (6.6 and 10.8 years) were tested for reweighting within each group and for group differences. Children with DCD reweight touch and vision at a later age (10.8 years) than their TD peers (4.2 years). Children with DCD demonstrate a weak visual reweighting, no advanced multisensory fusion and phase lags larger than those of TD children in response to both touch and vision.

**Conclusions/Significance:**

Two developmental perspectives, postural body scheme and dorsal stream development, are provided to explain the weak vision reweighting. The lack of multisensory fusion supports the notion that optimal multisensory integration is a slow developmental process and is vulnerable in children with DCD.

## Introduction

Children with Developmental Coordination Disorder (DCD) demonstrate motor coordination substantially below what is expected for their chronological age and measured intelligence. DCD affects as many as six in 100 school-age children [1], making it a leading developmental movement disorder. This poor motor coordination, commonly involving posture [2]–[5], upper extremity [6]–[8], and also cranial motor control [9]–[10], interferes with their activities of daily living and/or academic achievement. Studies have demonstrated multiple mechanisms underlying the poor coordination, with known deficits in timing [11]–[12] and force generation [12], as well as the sensory processing [13]–[16] critical to appropriate motor responses. Here we focus on children with DCD with recently developed techniques that not only characterize deficits in processing a single sensory stimulus, but also the integration of multiple sensory stimuli for flexible and stable postural control. The issue of multisensory integration is of great interest to those studying developmental disorders as its deficit is commonly suggested in children with various types of developmental disabilities [17]–[19], including children with DCD [3]–[5], [14]–[15], [20].

To integrate multisensory information, the nervous system continually changes its emphasis (i.e., weight) on visual, vestibular and somatosensory information to maintain upright stance as environmental conditions change [21]–[23]. This continuous change in emphasis (i.e., reweighting) requires complex computations directly demonstrated in animal neurophysiological recordings (for review see Stein et al., [24]). In human postural control, the complex computation underlying multisensory integration can be quantified by multisensory reweighting, an adaptive process in which the central nervous system down-weights unreliable sensory stimuli while simultaneously up-weighting more reliable sensory stimuli [23], [25]–[27]. To quantify sensory weights, one common experimental technique is to have subjects stand within a visual “moving room” [28]–[30]. The walls of the laboratory move but the floor that the subject stands upon remains motionless, creating conflicts between vision and the other senses (e.g., proprioception and the vestibular sense). The visual stimulus produced by very slow and small movements of the walls is similar to that observed during postural sway, leading to a strong coupling between postural sway and the visual movement. The coupling strength (i.e., weight) can be measured by gain, the magnitude of the postural response divided by the visual stimulus magnitude at the frequency of wall movement [22]–[23]. As the amplitude of the visual surround increases, the nervous system can no longer maintain a high gain (i.e., strong coupling) without the individual eventually being forced beyond their stability limits of postural sway. As a result, vision gain becomes smaller as visual scene movement increases in amplitude, signifying down-weighting [22]–[23]. The de-emphasis (i.e., down-weighting) on vision is generally accompanied by the nervous system’s greater reliance (i.e., up-weighting) on other modalities to estimate self-motion [22], [26], [31]. Such amplitude-dependent gain changes (i.e., smaller gain to larger amplitude) are observed in typically developing (TD) children [29]–[30] but not in newly standing infants [28]. Young infants maintain strong responses to increased visual scene movement and often fall [28], possibly due to less effective reweighting [25].

The ability to empirically quantify reweighting may significantly improve our understanding of the developmental onset of human multisensory integration, which has been previously estimated to occur in early infancy for non-postural tasks, much earlier than in animals (for review, see Stein et al. [24]). Stein et al. attribute the discrepancy between animal and human multisensory integration development to different experimental techniques and underlying assumptions. Specifically, human developmental studies often lack rigorous quantification to address the complex computation required to combine multiple sources of information to obtain a precise estimate [24], [32].

Multisensory reweighting protocols may also advance our understanding of multisensory integration development in children with developmental disabilities compared to TD children. In children with DCD, multisensory integration deficits have been proposed as a possible mechanism for poor postural control in these children [3]–[5], [20], however, we argue that such deficits are insufficiently characterized. Previous studies have either manipulated sensory stimuli in an all-or-none manner (e.g., open or close eyes) [3]–[5], [20] or created conflicts between sensory stimuli [3], [20]. The results generally show that children with DCD have significantly poorer postural control than their TD peers, particularly when the somatosensory information is unreliable (e.g., standing on compliant foam or moving surface) [4], [20]. Consistent with these studies, we found that children with DCD use enriched haptic information (i.e., lightly touching a stationary surface) less effectively than their TD peers, while relying more on vision [5], similar to the findings of Deconinck and colleagues [4]. Conflicting sensory information is especially challenging for children with DCD [3]. These studies in which sensory reweighting is not quantified suggest that multisensory integration may play an important role in postural deficits in children with DCD.

A reweighting deficit to single visual stimulus has been demonstrated by Wann et al. [29] using a “moving room” with changing room amplitude. In their study, Wann and colleagues found that those children with DCD who pass a clinical balance test demonstrated amplitude-dependent gain changes similar to their age-matched controls; whereas those children with DCD who had poor balance showed visual gain changes similar to younger children. These results suggest that compromised sensory reweighting may be one underlying mechanism for the postural deficits observed in children with DCD. However, the moving room paradigm quantifies reweighting in only one sensory system; vision. To our knowledge, no study has empirically demonstrated reweighting in a multisensory context for children with DCD.

In the current study, we address these issues in children with DCD by implementing a recently developed experimental protocol in which subjects view a moving visual scene while lightly touching a moving bar with the fingertip. The visual scene and touch bar oscillate at difference frequencies and their movement amplitudes are varied to reveal how an individual’s weighting of different sensory stimuli depend on stimulus movement amplitudes [23], [33]. Studies in adults show that the gain to each individual sensory modality depends not only on that specific modality’s amplitude (i.e., intra-modal reweighting) but also to the amplitude of the other coexisting modalities (i.e., inter-modal reweighting). We implemented this protocol to investigate how TD children develop the ability to use multisensory information for postural control [25]. We demonstrated that children as young as 4 years of age are able to reweight to both vision and touch information and the amount of reweighting increased with age indicating a better adaptive ability in older children. Moreover, the fusion of touch and vision sensory information, as indicated by inter-modal reweighting, was observed only in the 10-year-old children. These results are in agreement with the notion that multisensory development is a process of achieving optimal multisensory fusion [34]. That is, even though young children can use sensory information from multiple sources, their optimal integration is not achieved until middle childhood [32], [34]–[35]. With increased age in childhood, the adaptive multisensory reweighting may facilitate fusion of multisensory information in a statistically optimal way [36]–[37] to produce a robust percept [38] and disambiguate conflicting sensory information for perception [38]–[39] and action (e.g., postural control [40]).

To our knowledge, there are no studies that quantify multisensory reweighting for postural control and development in children with DCD. In this study, we implement an established multisensory reweighting protocol for postural control [23], [25], [33] in children with DCD from 6 to 11 years of age. We directly compare the multisensory reweighting statistically in children with DCD to a previously published dataset from TD children 4 to 10 years old [25]. Specifically, we ask two questions: 1) Can children with DCD reweight both touch and visual sensory information as previously observed in TD children [25]? And, 2) Do children with DCD show advanced multisensory fusion (i.e., inter-modal reweighting) as previously observed in TD children about 10 years of age [25]?

## Materials and Methods

### Ethic Statement

This study followed the principles of Declaration of Helsinki for human subject protection, and the protocol was approved by the Internal Review Board at the University of Maryland, College Park. Written informed consent from the parents and written assent from the children were acquired for each participant.

### Subject Compensation

Each child was paid a nominal sum for his or her participation.

### Subjects

The data on TD children were from a previously published study [25] that characterized the developmental profile of multisensory integration for postural control. There were forty-one TD children ranging in age from 4.2 to 10.8 years old (21 boys, 20 girls; mean ± std  = 7.5±1.9 years). Their motor development was considered typical as evaluated by the Movement Assessment Battery for Children (MABC) [41] if they had a score above 20^th^ percentile.

For the current study, a total of sixty-two children with motor coordination concern were recruited through referral from specialists (pediatricians, therapists or educators), brochures and other advertisements. After acquiring informed consent from the parents and assent from the children, all children underwent a double-blinded screening process in which a developmental pediatrician performed a clinical examination including a medical history and a neurodevelopmental examination using the Physical and Neurological Examination for Soft Signs (PANESS) [42]. The physician independently determined if a child met the DSM-IV DCD diagnosis criteria [1]. A physical or occupational therapist from the research team independently tested the child with the MABC. Both a DCD diagnosis from the physician and a MABC performance less than the 5^th^ percentile were required to be included in the DCD group. We followed the recommendation for DCD research [43] and chose a score on the MABC less than the 5^th^ percentile as the cut-off point for identifying those with DCD. The cognitive ability of all the eligible children was within normal limits as assessed by Woodcock-Johnson Revised Cognitive Ability Early Development Scale [44]. After all screening, twenty-six children were eligible and 21 of these were willing to participate in the posture study that was conducted on a different day than the screening tests. Twenty children ranged from 6.6 to 11.8 years of age completed the posture test (17 boys, 3 girls; mean ± std  = 9.2±1.6 years) (see Table 1). The only child who did not complete the posture test was the youngest child tested (5.6 years) who could only perform a few trials due to the inability to follow instructions. For children with DCD completing the posture test, their MABC total impairment scores ranged from 13.5 to 36 out of a maximum possible score of 40 (mean ± std  = 22.1±6.4). The impairment score on the balance subsection ranged from 0 to 14 out of a maximum possible score of 15 (mean ± std  = 6.7±4.1). Note that we included children with DCD whose posture impairment scores were low (lower impairment score indicates better balance) so long as they met both inclusion criteria (i.e., physician’s diagnosis and MABC less than 5^th^ percentile). We justify our inclusion of these children who did not show obvious balance impairment (i.e., assessed behaviorally by the MABC balance subsection) because we are interested in determining if our multisensory reweighting paradigm can detect subtle balance deficits under a complex and dynamic environment.

**Table 1:** Age, sex and MABC performance for children with DCD.

	Test Age (years old)	Sex	MABC Total Impairment Score	MABC Balance Impairment Score	MABC percentile
	6.6	M	17.5	6.0	1
	7.0	M	32.0	11.5	<1
	7.1	F	20.5	12.5	<1
	7.4	M	23.0	12.5	<1
	7.8	M	15.5	5.5	3
	7.8	M	19.5	2.5	<1
	8.3	F	15.0	0.0	<3
	8.7	M	15.0	3.0	3
	8.8	M	19.0	0.5	<1
	9.3	M	13.5	6.0	5
	9.5	M	17.5	8.5	1
	9.7	F	24.5	8.5	1
	9.9	M	27.5	8.5	<1
	10.0	M	21.5	2.5	<1
	10.4	M	24.0	6.5	<1
	10.8	M	36.0	11.0	<1
	10.9	M	25.5	6.5	<1
	11.3	M	15.0	3.0	3
	11.4	M	28.0	5.7	<1
	11.8	M	31.0	14.0	<1
Mean	9.2		22.1	6.7	
Std Dev	1.6		6.4	4.1	

A high impairment score in the total (max.  = 40) and the Balance sub-section (max.  = 15) reflects poor motor ability. The percentile refers to the percentile ranking derived from the MABC scoring of the overall impairment score.

### Task

**Figure 1:**
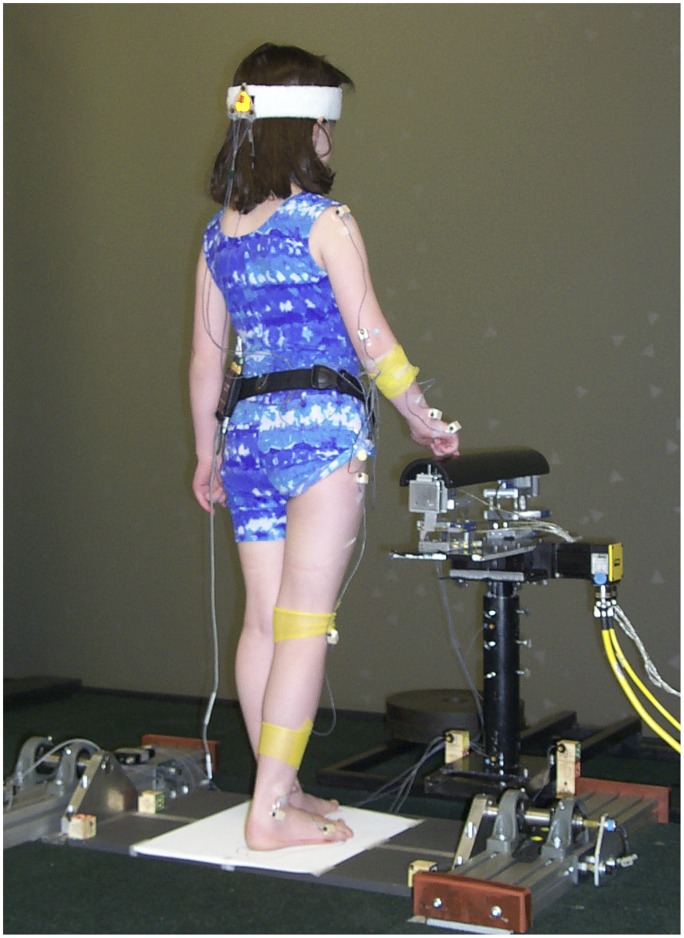
Experimental set-up showing a child performing the multisensory posture task. The child stood in a modified semi-tandem stance looking at a front screen with random dots projection (not shown due to room illumination) while touching a bar lightly without triggering an auditory alarm. Both the visual display and the touch bar moved in the mediolateral direction simultaneously but with different frequencies. Markers were placed on the head and on the right side of the arm, and lower leg to track postural kinematics.

Children were asked to stand in a modified semi-tandem stance with the inner edges of the feet aligned in the sagittal plane. They were given several opportunities to try the stance and decide which foot to place in front of the other. Once they decided on the preferred stance, the feet positions were traced on the supporting surface so that the same stance configuration could be kept throughout the test. The children were instructed to look at a front screen (for details see “Visual display” section) and touch a bar lightly with their index finger without triggering an auditory alarm (for details see “Touch bar” section) while maintaining their balance. Practice was provided to familiarize the children with maintaining their stance, looking at the front screen, and avoiding triggering the alarm when touching the touch bar. After the children performed each subtask correctly, we asked them to keep the modified semi-tandem stance while quietly looking at a front screen with their right index finger lightly touching a bar (Fig. 1).

### Apparatus

#### Touch bar

A touch bar (diameter: 4.4 cm, length: 45.7 cm) with a PVC convex surface was designed to be “touchable” without being “graspable” by the children. The touch bar was positioned level with the child’s right hip height in the frontal plane. The right elbow position was about 135° when the index finger lightly contacted a fixed point on the touch bar. To ensure that the children used the touch bar primarily for sensory information, a threshold was set at 1 Newton vertical touch force. An auditory alarm sounded if the child pressed the touch bar harder than the threshold level. Children were instructed not to trigger the auditory alarm. In situations where the alarm went off, they were asked to maintain their index finger in contact with the bar but reduce the force applied until the alarm stopped. Touch force was monitored during data acquisition to ensure that the child touched the bar throughout the trial. Data collection was stopped if the child lifted the index finger off the bar and the trial was repeated. Most children only needed a few practice trials before they were able to maintain light finger touch throughout the test.

To test how children use touch information for postural control, the bar was controlled by a servo-motor and moved in the medial-lateral direction at 0.28 Hz with specified amplitudes (details see “Experimental design” section). The children were not informed of the touch bar’s movement. The touch bar oscillation is referred to as Tdrive.

#### Visual display

The visual displays for the two groups of children were somewhat different. However, the most important features (i.e., wide visual field, no projection in the central visual field to reduce aliasing effects, and display patterns) were comparable across the two visual display setups. In our previous study with the TD children [25], the visual display was a front screen, 250 cm wide and 100 cm high. Children stood 40 cm away from the middle of the screen and wore goggles to keep the screen edges from being visible. The visual range was approximately 100° high and 120° wide. A total of 100 random triangles were rear-projected on the screen with black background when the room was dark. Each triangle was about 0.2°×0.2°×0.2° in diameter when it was projected statically on the screen directly in front of the subject at the subject’s eye height. No triangles were projected in a circular area (about 10° visual range) centered directly in front of the subject at eye height to reduce the aliasing effects most noticeable in the foveal region.

For the test of children with DCD, the visual display consisted of three screens (each 305 cm wide and 244 cm high) attached at right angles that surrounded the subject (one in front, one to the right, and one to the left). Children stood halfway between the left and right screens, facing the front screen at a distance of ∼107 cm. The visual range was approximately 100° high and 110° wide (compared to 100° high and 120° wide used for TD children). Children did not wear goggles as in the previous visual display setup because the edges of the front screen were not visible to the subject as the background of the adjacent screens was black and the room was dark. Each screen had 500 white triangles rear-projected onto it with the triangle positions and orientations randomized. Each triangle size was about 0.2°×0.2°×0.3° in diameter when it was projected statically on the front screen directly in front of the subjects at their eye height. Similar to the previous setup, triangles were not projected in a circular area (30-cm radius, ∼15° visual range) of the front screen centered directly in front of the subject at eye height.

For the one-screen setup, in order to test how children use visual information for postural control, the projected visual display of the front screen oscillated in the medial-lateral direction at 0.2 Hz with specified amplitude (details see “Experimental design” section). For the experiment with children with DCD using a three-screen setup, CaveLib software (Fakespace) was used to generate a virtual moving visual scene consisting of three walls attached at right angles that coincided with the screens when the visual scene was at its initial resting position. The display on each screen was varied with time to simulate medial-lateral oscillation of the visual scene at 0.2 Hz with specified amplitude, assuming a fixed perspective point at the average position of the subject’s eyes. As a result, the triangles on the front scene translated in the medial-lateral direction as in the one-screen setup. The projected triangles on the two side screens contracted toward or expanded away from the point on screen directly lateral to the perspective point. For example, translation of the front-screen display to the right was accompanied by contraction of the right-screen display and expansion of the left-screen display. Note that the contraction and expansion of the side screens displays still created medio-lateral translational optic flow to the subjects because they were looking forward at the front screen. The children were not informed of the visual display’s movement. This visual display oscillation at 0.2 Hz was referred to as Vdrive. Note that the Vdrive frequency was different than the Tdrive frequency of 0.28 Hz, which allowed us to independently measure the postural responses to both types of motion.

### Kinematic Recording

For the previously published study with TD children [25], postural sway was recorded by a 3D ultrasound position tracking system (Logitech, Inc) at a sampling rate of 50.33 Hz. Ultrasound markers were attached to back of the head and approximate center of mass (CoM). For the test of children with DCD, postural responses were recorded by Optotrak position sensors (Northern Digital, Inc., Waterloo, ON, CA) sampled of 60 Hz. Markers were placed at ankle (lateral malleolus), knee (lateral tibial tuberosity), hip (greater trochanter), and shoulder (acromion) to the right side of the body. CoM trajectories were estimated using a three-segment model [45] based on the markers’ trajectories. Three markers arranged in equal-side-triangle configuration were attached to back of subject’s head (occipital protuberance) and the head trajectories (Head) were calculated from these three markers. Only medial-lateral postural responses from Head and CoM were reported because the drives oscillated in the medial-lateral direction. Markers were also attached to the right elbow and wrist to monitor on-line if the child’s finger lifted from the touch bar.

### Experimental Design

The experimental designed is based on a previous study [23] and had been implemented in young adults [23], elderly [33] and TD children [25]. To test how individuals integrate both touch and visual information for static postural control, the touch bar and visual scene positions were simultaneously oscillated during a trial. Tdrive and Vdrive frequencies were chosen with an approximate ratio of √2 to avoid common low-order harmonics. To investigate the multisensory reweighting (i.e., amplitude dependent gain changes), amplitudes of the two sensory stimuli were systematically manipulated. Specifically, the oscillation amplitude of one modality was kept constant across some of the test conditions while the amplitude of the other modality was systematically manipulated. A total of five amplitude pairs were used (T_8_V_2_, T_4_V_2_, T_2_V_2_, T_2_V_4_ and T_2_V_8_; where subscripts indicate mean-to-peak amplitude in mm in the medial-lateral direction). For the first three amplitude pairs (i.e., T_8_V_2_, T_4_V_2_, and T_2_V_2_), Vdrive amplitude was held constant at 2 mm while the Tdrive amplitude changed from 8 mm to 4 mm to 2 mm. The same principle applied to the three conditions (i.e., T_2_V_2_, T_2_V_4_ and T_2_V_8_) where Tdrive amplitude was the same while Vdrive amplitude changed. Postural responses to Tdrive and Vdrive (i.e. touch gain and vision gain, respectively) across these five conditions were used to examine the amplitude-dependent gain changes, which we interpreted as sensory reweighting as supported by modeling work [27], [31] and as proposed by others [23], [25], [27], [31], [33], [46].

Each trial was 100 seconds long and three repetitions were tested for each condition (a total of 15 trials). Five conditions were grouped into one block and randomized within a block. Breaks were provided as the child requested. The breaks requested by the children ranged from every trial to every 3 trials. Every child but one with DCD completed the postural test with one visit to our laboratory. The time it took to finish the test ranged from one and a half to three hours. One child (a 7.4 year-old boy in the DCD group) required more than one test session because it took a long time for him to get used to the markers attached to him. This child completed the postural test in the second test session smoothly taking about two and a half hours. No child lost balance during the test.

### Analysis

#### Preprocessing

**Figure 2:**
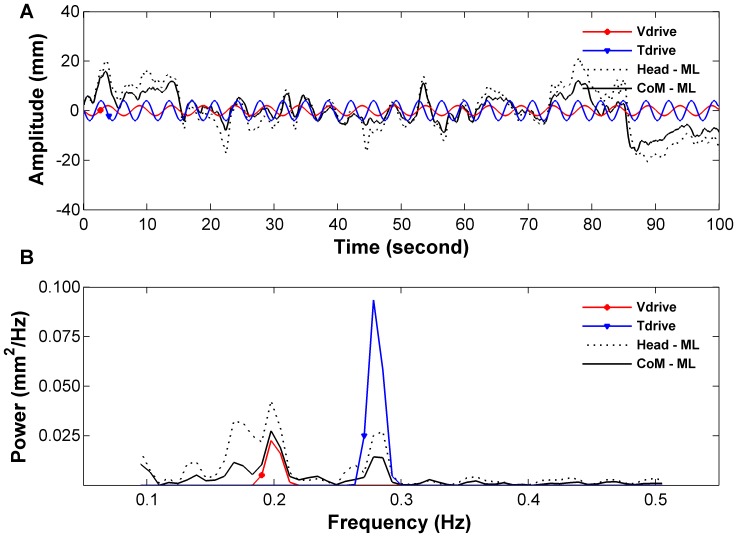
Exemplar of drives and sway from a T_4_V_2_ trial of a child with DCD. Subplot A shows the time series of the trajectories, and subplot B shows their power spectrum in the frequency domain. Note that the Tdrive and Vdrive oscillated at different frequency (0.28 and 0.2 Hz respectively).

Customized MATLAB™ (Mathworks, Natick, MA, USA) programs were used for data analysis. Raw Head and CoM postural response in the medial-lateral direction, the Tdrive and the Vdrive were zero meaned and passed through a zero-phase low-pass filter consisting of a forward-reverse cascade (i.e., implemented using MATLAB’s filtfilt function) of a 4^th^-order low-pass Butterworth filter with a cutoff frequency of 5 Hz. Figure 2 is an exemplar of the time series of the two drives (i.e., Tdrive and Vdrive) and postural sway of the Head and CoM for a T_4_V_2_ trial (Tdrive: 4 mm, Vdrive: 2 mm) (Fig. 2 A), and an exemplar of the spectral plots of postural sway and sensory stimuli (Fig. 2 B).

#### Frequency response functions (FRFs) with gains and phases

**Figure 3:**
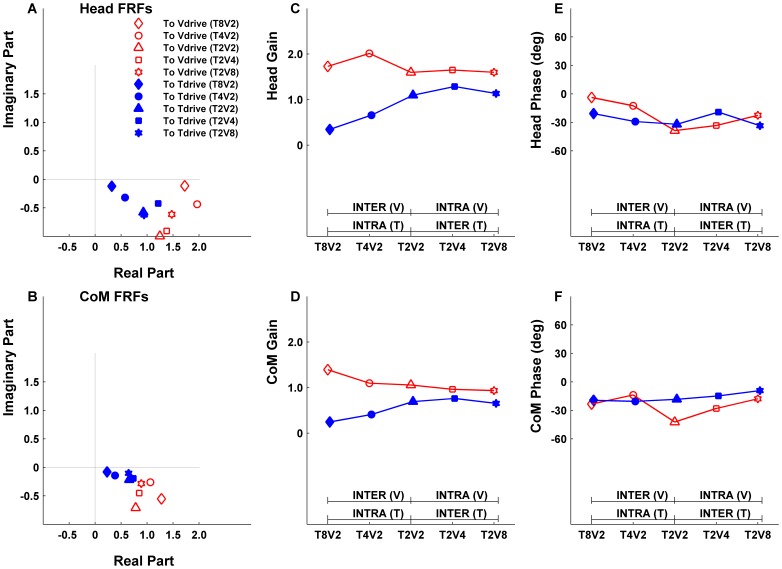
Exemplar of averaged frequency response functions (FRFs), gains and phases. Results are averaged across three trials for each condition from all five test conditions in a 7.6-year-old child with DCD. Subplots in the first column show the FRFs in the complex plane for the Head (A) and CoM (B). The distances from the FRFs to the origin are the gains and they are plotted in the middle column subplots for Head gains (C) and CoM gains (D) to Tdrive (filled markers) and Vdrive (open face markers). Intra-modal reweighting refers to gain changes due to the change of a specific modality’s amplitude. For example, symbol such as |- INTRA (T) -| indicates that touch gain intra-modal reweighting is evaluated across T_8_V_2_ and T_2_V_2_ conditions. Similarly, |- INTRA (V) -| indicates that vision gain intra-modal reweighting is evaluated across T_2_V_2_ and T_2_V_8_ conditions. Inter-modal reweighting refers to gain changes due to the change of a coexisting modality’s amplitude. For example, symbol |- INTER (T) -| indicates that touch gain inter-modal reweighting is evaluated across T_2_V_2_ and T_2_V_8_ conditions while |- INTER (V) -| indicates that vision gain inter-modal reweighting is evaluated across T_2_V_2_ and T_8_V_2_ conditions. A significant gain difference between conditions indicates reweighting. This child demonstrates intra-modal touch reweighting. The angle between the FRFs and the positive real axis are phases and they are plotted in the right column subplots for Head phase (E) and CoM phase (F). Note that phases are all negative indicating a phase lag of postural response to drives.

Postural responses to the drives were measured by the frequency response functions (FRFs) at the driving frequencies (i.e., FRF at 0.28 Hz for Tdrive, FRF at 0.2 Hz for Vdrive). One FRF was calculated for each segment’s postural sway (i.e., Head or CoM) to each drive (i.e., Tdrive or Vdrive), thus a total of four FRFs were calculated for each trial (Head to Tdrive, Head to Vdrive, CoM to Tdrive and CoM to Vdrive). The FRF is a complex number with gain (absolute value of the FRF) representing response amplitude divided by stimulus amplitude and phase (argument of the FRF) representing the temporal relationship between postural sway and the drives. A negative phase indicates that the postural response lags behind the given drive. The FRFs were computed for the last 75-second segment of the time series. The first 25-second segment was not analyzed to exclude transient postural responses to the drives’ onset. Welch’s method with 25-second windows (least common multiple of the two drives’ periods) and 50% overlap was used to calculate FRFs. FRFs were averaged across three trials for each condition for each subject. Figure 3 is an exemplar of the averaged FRFs, gains and phases across five conditions of a 7.6-year-old child with DCD.

#### Statistical analysis

For all statistical tests, condition was treated as a within-subject repeated factor, and age and group as between-subject non-repeated factors; *p<*0.05 was considered significant and *p<*0.1 was considered marginally significant.

#### Fits of gain and phase

For each group (TD and DCD), segment (Head and CoM) and sensory drive (Tdrive and Vdrive), we used the method of Bair et al. (2007) [25] to fit gain and phase by constant, linear, quadratic and cubic functions of age. Based on the comparison of these fits (see “Results” section), we chose to use linear fits in our statistical analysis of group and condition effects.

To analyze group and condition effects, a separate nonlinear multivariate regression analysis was conducted for the postural responses of each segment to each drive. For each group *g* and condition *c*, we used the FRFs *H*_gcs_ for subjects *s = *1,…,*n_g_* to fit gain *γ*_gc_(*a*) and phase *ϕ_gc_*(*a*) simultaneously as linear functions of age *a*:
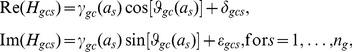
(1)where *a_s_* is the age of subject *s*. The linear fits *γ_gc_*(*a*) and phase *ϕ_gc_*(*a*) were chosen to maximize the model’s concentrated log-likelihood [47] under the assumptions that the errors (*δ_gcs_*, *ε_gcs_*) for different subjects *s* had a multivariate normal distribution. Fits were subject to the constraint that 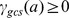 over the age range of the given group. Note that although gain and phase were fit by linear functions of age, our regression model (1) is nonlinear because the real and imaginary parts of the FRFs are nonlinear functions of gain and phase. We chose this nonlinear approach for two reasons (Bair et al., 2007) [25]. First, estimates of the real and imaginary parts of FRFs, unlike estimates of gain, are unbiased [48], consistent with the way that random variation is specified in model (1). Second, model (1) naturally incorporates the fact that phase is a circular variable.

**Figure 4:**
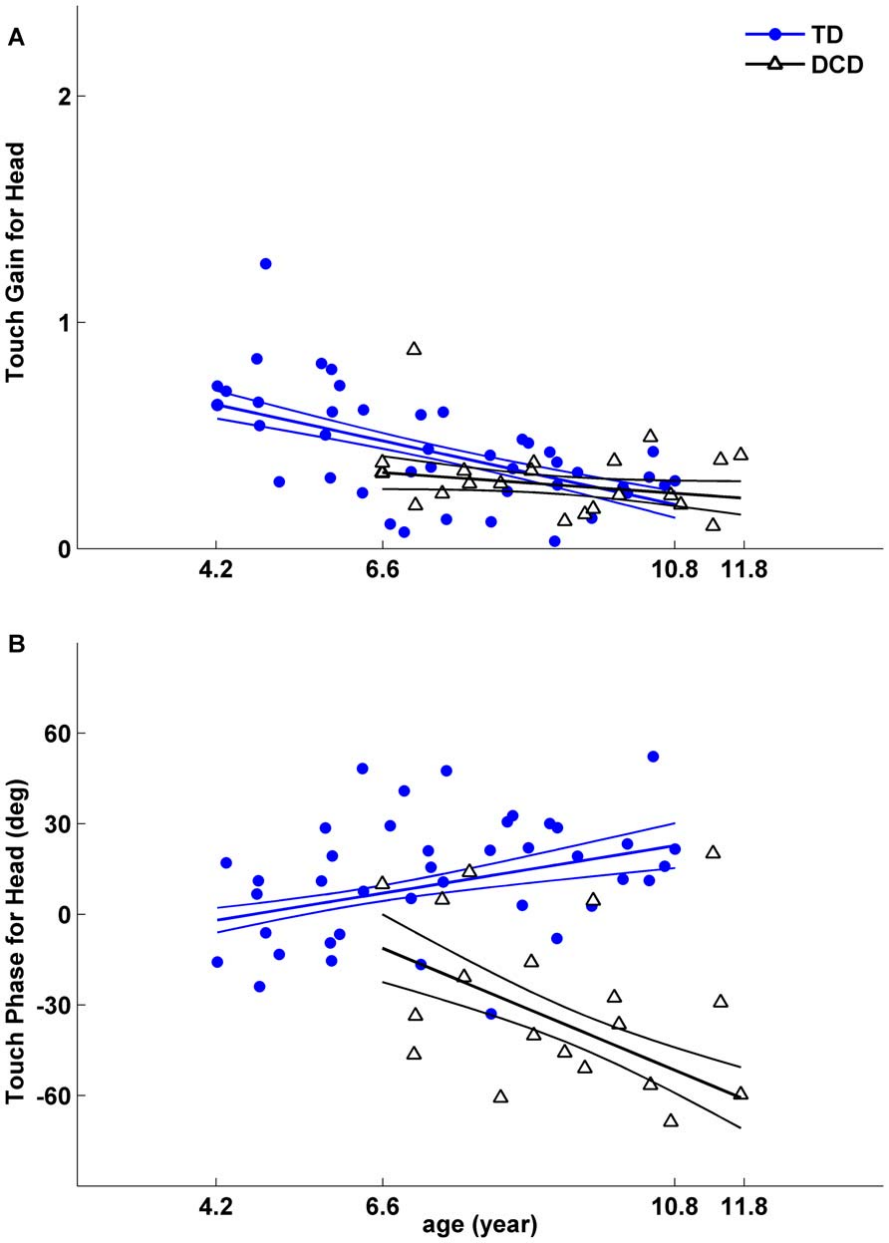
Linear fit of gains and phases as a function of age. The linear fit is for each group of children for one condition. Here the gains (A) and phases (B) are from Head responses to Tdrive in a T_8_V_2_ condition. Gains (A) and phases (B) from 41 TD children (filled markers) and from 20 children with DCD (open face markers) with fitted gain lines and fitted phase lines are plotted with associated standard errors calculated from bootstrapping. The age range used for model fitting is from 4.2 to 10.8 years of age for the TD children and from 6.6 to 11.8 years for children with DCD. We evaluate the model fitting for both groups at a lower comparison age of 6.6 years and an upper comparison age of 10.8 years as shown in the following Figures 5, 6, 7 and 8. Note that children with DCD showed delayed phase of postural response to Tdrive comparing to their TD peers.

Figure 4 shows an exemplar of one such model fit. Here linear gain and phase functions were fit based on the FRFs for Head in response to Tdrive for each subject and each group in a T_8_V_2_ condition. The linear fitting was performed for each group with different age ranges. That is, the model fitting for the TD group was from 4.2 to 10.8 years old, and for the DCD group from 6.6 to 11.8 years old. Although the models were fitted for all the data collected for each group, to make a direct comparison between two groups, we chose the lower comparison age to be 6.6 years old and the upper comparison age to be 10.8 years old to avoid extrapolations of the fitted data. Note that because the fitted gain and phase functions were linear, they could be fully specified by their values at the lower and upper comparison ages.

#### Examples of intra-, inter-, and total reweighting

This protocol investigates whether reweighting is sensitive to changes in the modality that changes amplitude as well as if reweighting is sensitive to the modality that remains at a constant amplitude. For example, *intra-modal reweighting* is an increase in touch gain from T_8_V_2_ to T_2_V_2_ or a decrease in vision gain from T_2_V_2_ to T_2_V_8_ (i.e. gain of the response to a modality is sensitive to the change in the amplitude of that modality). Whereas *inter-modal reweighting* is an increase in touch gain from T_2_V_2_ to T_2_V_8_ or a decrease in vision gain from T_8_V_2_ to T_2_V_2_ (i.e. gain of the response to a modality is sensitive to the change in the amplitude of another simultaneously presented modality). Inter-modal reweighting to a constant amplitude modality due to another coexisting modality with changing amplitude is interpreted as fusion of the two sensory modalities. *Total reweighting* (sum of intra- and inter-modal reweighting) is an increase in touch gain or a decrease in vision gain from T_8_V_2_ to T_2_V_8_. We included the analysis of total reweighting because it increased our ability to detect reweighting if both intra- and inter-modal reweighting were small.

#### Hypothesis testing

Bootstrap tests were used to test various hypotheses concerning the fitted gain and phase values at the two comparison ages. Bootstrap tests were performed by fixing the ages of the subjects and resampling the residuals of the fits [49]. To test whether a vector *θ* of parameters is different than 0, we used a method based on the normal approximation (Hall 1992, p. 159, [50]). We computed the statistic 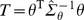 for the original data and for 10^4^ bootstrap resamples of the residuals at the first level, where 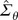 is the bootstrap estimate of the variance-covariance matrix of *θ* based on 10^3^ nested bootstrap resamples at the second level. We computed the *p*-value as the fraction of resamples yielding values of *T* greater than the value of *T* for the original data.

A separate analysis was performed for each segment and drive. For each group, we first tested for an overall dependence on age using data from all five conditions. Next, we performed a detailed analysis of group, age and condition effects using data from conditions in which the visual-scene and touch-bar motions were at their highest or lowest amplitudes: T_8_V_2_, T_2_V_2_ and T_2_V_8_. For each group, we performed nine tests involving gain. For each pair of conditions, we tested for a condition effect at the lower comparison age, a condition effect at the upper comparison age, and an age-by-condition interaction. We controlled the family-wise type I error rate for the nine tests by adjusting p-values using a closed testing procedure [51]. We also performed nine tests comparing gain between groups. For each pair of conditions, we tested for a group-by-condition interaction at the lower comparison age, a group-by-condition interaction at the upper comparison age, and a group-by-condition interaction. Again, a closed-testing procedure was used to control the family-wise error rate. The method used to analyze gain effects was also applied to phase.

## Results

The patterns of group difference (or lack of difference) were essentially the same for both CoM and head (see details below). However, we chose to present both results for two reasons. First, there are very limited data pertaining to sensory reweighting for postural control either in TD children or in children with DCD. These previous studies reported either only CoM or head results. We considered it useful to present both results so that the gain and phase response values (i.e., not just the patterns of group difference) are available in the literature. Second, we presented both CoM and head results to be consistent with our previously report format in TD children [25]. As for those children with DCD with low MABC posture impairment scores (i.e., better balance), their reweighting patterns to Vdrive and Tdrive were not qualitatively different from those children with DCD with high impairment score supporting the view that our multisensory reweighting paradigm can detect subtle balance deficits even in these children. Therefore, we included these children in the analysis as planned.

For the previously published study with TD children [25], a linear model of the dependence of gain and phase on age fit the data significantly better than a constant model, indicating that postural responses changed with age in TD children (Head to Tdrive: Wilks’ Λ = 0.49, *F*_10,30_ = 3.08, *p = *0.008; Head to Vdrive: Λ = 0.47, *F*_10,30_ = 3.32, *p = *0.005; CoM to Tdrive: Λ = 0.41, *F*_10,30_ = 4.26, *p = *0.001; CoM to Vdrive: Λ = 0.52, *F*_10,30_ = 2.76, *p = *0.015). Quadratic and cubic models were also not significantly better than the linear model (p>0.05). We concluded that the model with linear gain and phase functions provided an adequate description of age-dependent changes for TD children.

**Figure 5:**
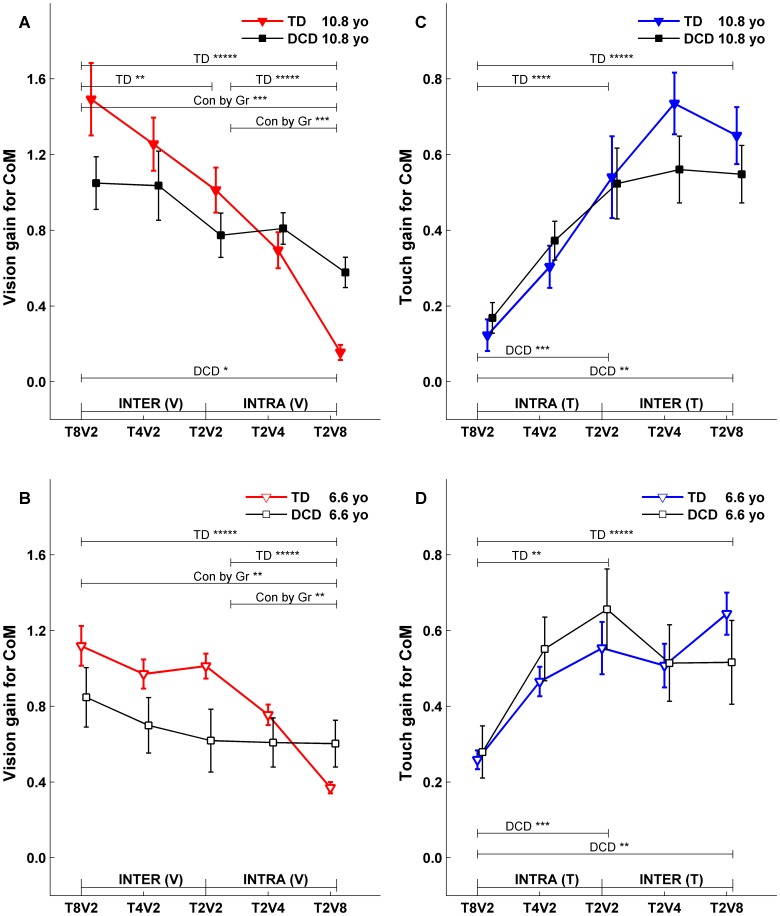
Fitted CoM gains at upper (10.8 years) and lower (6.6 years) comparison ages. Each fitted gain with its corresponding standard error was extracted from a linear model fit (exemplified in Figure 4A) for the specified condition, segment, sensory drive and comparison age. Fitted gains from 5 conditions were plotted in a subplot to summarize intra-, intermodal and total reweighting for each group (TD: triangle marker; DCD: square marker) and to contrast group difference. Subplot (A) is the CoM gain to Vdrive at upper comparison age, (B) is the CoM gain to Vdrive at lower comparison age, (C) is the CoM gain to Tdrive at upper comparison age, and (D) is the CoM gain to Tdrive at lower comparison age. Symbol such as |- INTER (V) -| indicates which two conditions are used to evaluate if there were inter-modal reweighting to Vdrive. Similar symbols are used for intra-modal reweighting and for reweighting to Tdrive. * indicates significant condition effect (***** for p<0.0001, **** for p<0.001, *** for p<0.01, ** for p<0.05, and * for marginal significance with p<0.1) for each group (labeled as TD, DCD respectively) or for group comparison (labeled as “Con by Gr”, indicating condition-by-group interaction). The solid bracket symbol indicates which two conditions are being compared. For example, the larger solid bracket is for the total reweighting between T8V2, and T2V8. The smaller bracket is for T8V2 & T2V2 condition pair or T2V2 & T2V8 condition pair.

**Figure 6:**
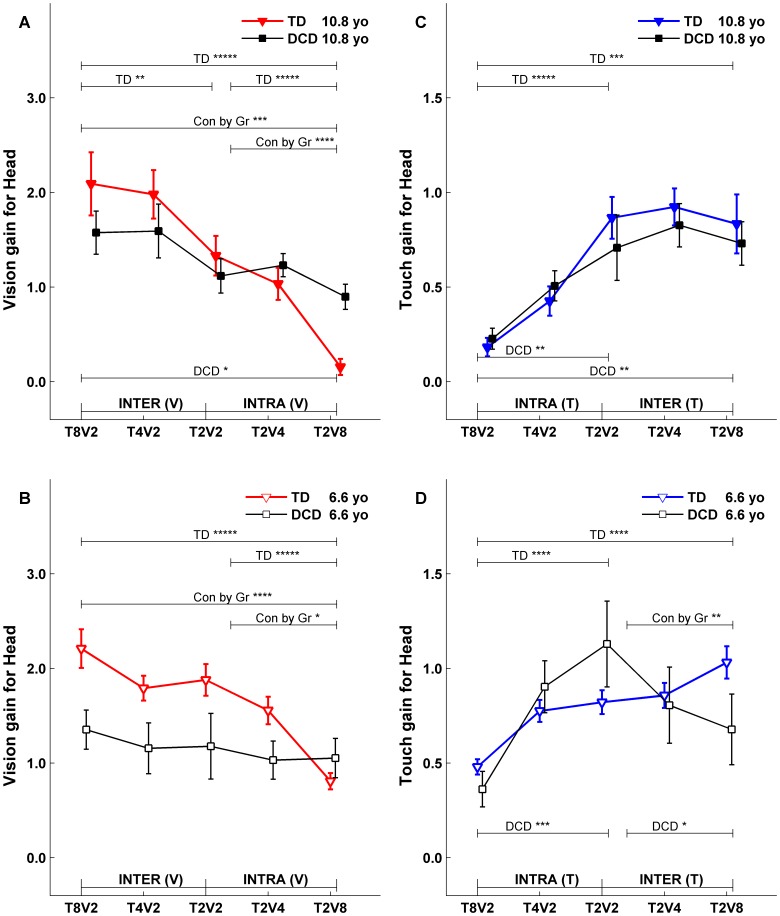
Fitted Head gains at upper (10.8 years) and lower (6.6 years) comparison ages. Symbol notations, legends for statistical significance are the same as in Figure 5.

**Figure 7:**
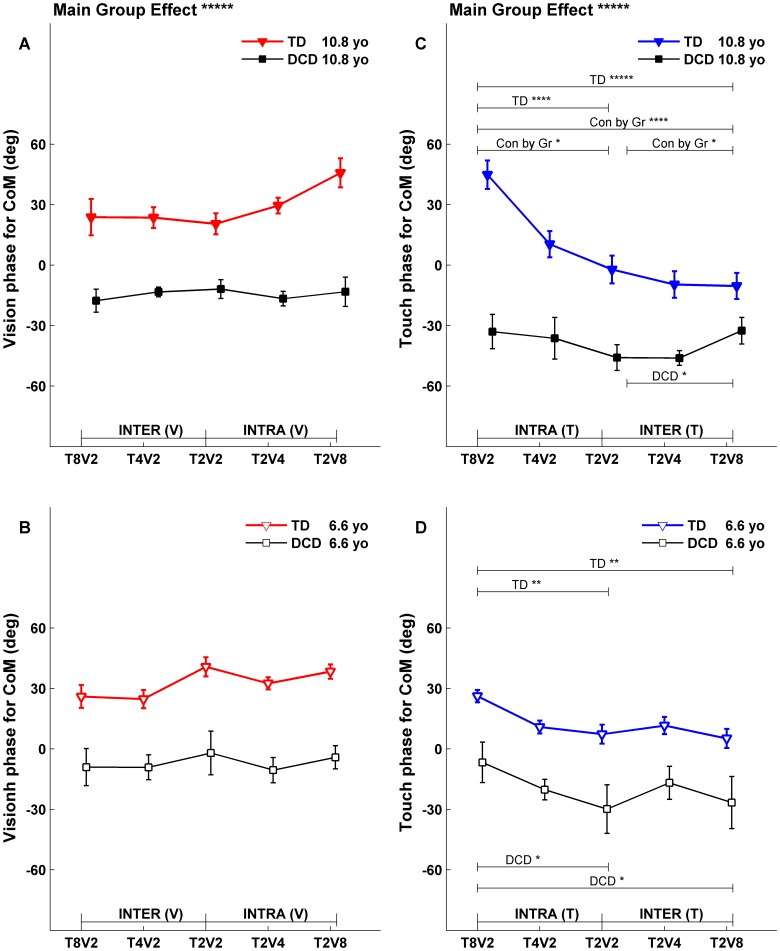
Fitted CoM phases at upper (10.8 years) and lower (6.6 years) comparison ages. Each fitted phase with its corresponding standard error was extracted from a linear model fit (exemplified in Figure 4B) for the specified condition, segment, sensory drive and comparison age. Symbol notations, legends for statistical significance are the same as in Figure 5.

**Figure 8:**
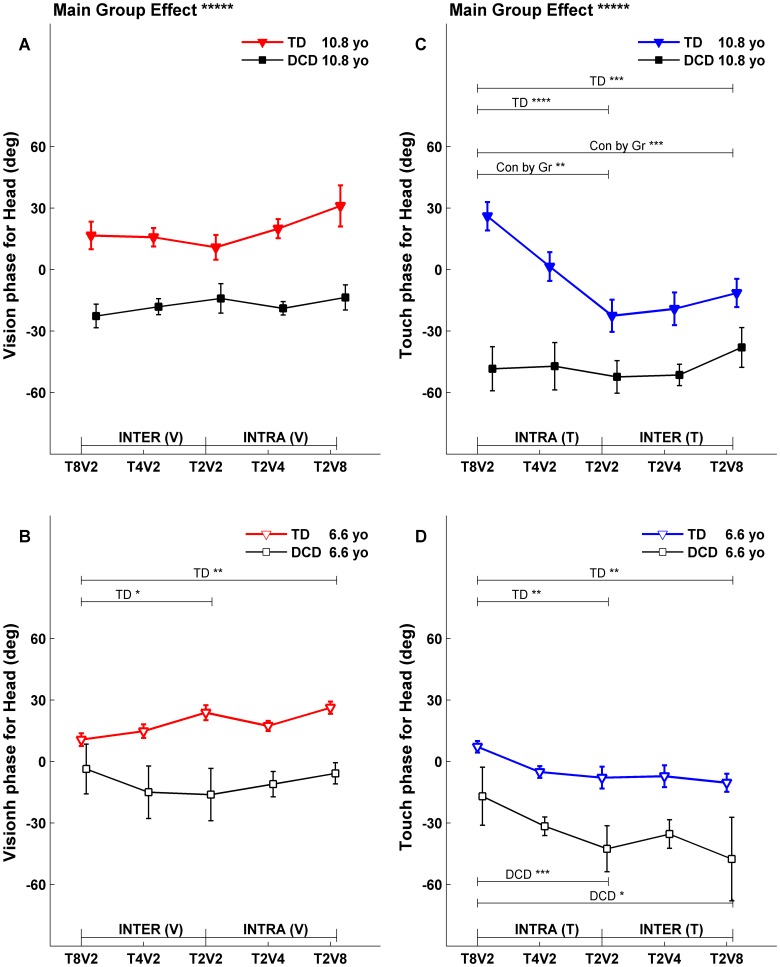
Fitted Head phases at upper (10.8 years) and lower (6.6 years) comparison ages. Symbol notations, legends for statistical significance are the same as in Figure 5.

Applying the method from Bair et al. (2007) [25] to the data from children with DCD, we found that the linear model fit the data significantly better than the constant model for their postural response to Tdrive, indicating that postural responses to Tdrive changed with age (Head to Tdrive: Wilks’ Λ = 0.21, *F*_10,10_ = 3.32, *p = *0.043; CoM to Tdrive: Λ = 0.17, *F*_10,10_ = 4.49, *p = *0.012). Quadratic and cubic models were not significantly better than the linear model (*p>*0.05). For postural response to the Vdrive, the linear model and higher order models did not fit the data significantly better than the constant model. However, to allow for a consistent presentation of results for both groups and both sensory drives, we chose to present the linear fits in all cases. Because of this choice to fit gain and phase as linear functions of age, the fitted values at the lower and upper comparison ages in Figures 5, 6, 7, and 8 completely specify the fitted functions across all ages.

To directly compare the fitted gains and phases between TD children and children with DCD, the fitted gain and phase functions were evaluated at the lower (6.6 years old) and upper (10.8 years old) comparison ages. The fitted gain lines evaluated at comparison ages across five conditions for both groups were plotted in Figure 5 for postural gain response of CoM, and in Figure 6 for postural gain response of Head. Similarly, the fitted phase lines evaluated at comparison ages across five conditions for both groups were plotted in Figure 7 for postural phase response of CoM, and in Figure 8 for postural phase response of Head. Each gain and phase value in Figures 5, 6, 7 and 8 is based on the FRFs of all subjects for corresponding group. When referring to Figures 5, 6, 7 and 8 for testing certain hypotheses, it is important to remember that each statement corresponds to an equivalent statement about the fitted gain (or phase) lines. For example, testing whether fitted gain values for two conditions are the same at the lower comparison age is equivalent to testing whether the corresponding two fitted gain lines intersect at the lower comparison age.

### Fitted Gains at Comparison Ages

For the gain responses (Figure 5 and 6), if subjects reweight to sensory stimuli across conditions, we expect vision gain (Figure 5A, B and Figure 6A, B) to decrease from left to right including *intra-modal reweighting* from T_2_V_2_ to T_2_V_8_, *inter-modal reweighting* from T_8_V_2_ to T_2_V_2_, and *total reweighting* from T_8_V_2_ to T_2_V_8_. Analogously, touch gains (Figure 5C, D and Figure 6C, D) are expected to increase from left to right. These three types of reweighting were tested at lower and upper comparison ages, and group-by-condition interactions tested whether reweighting differs between groups.

#### Gains to the Vdrive

For TD children, CoM to vision gain at the upper comparison age of 10.8 years (Figure 5A, filled triangles) showed evidence of total (p<0.0001), intra-modal (p<0.0001), and inter-modal (p<0.05) reweighting. At the lower comparison age of 6.6 years, TD children also showed significant CoM to vision gain of total (p<0.0001) and intra-modal (p<0.0001) reweighting (Figure 5 B, open face triangles). Thus, TD children reweight to the Vdrive while older TD children show the inter-modal visual reweighting indicating the ability to fuse multisensory information.

In contrast, children with DCD at the upper comparison age of 10.8 years (Figure 5 A, filled squares) only showed a marginal total visual reweighting. No visual reweighting was observable at the lower comparison age of 6.6 years (Figure 5B, open face squares). The difference in visual reweighting patterns between the two groups of children is supported by a significant Group by Condition interaction at the upper comparison age (Figure 5A) for total visual reweighting (p<0.01) and intra-modal visual reweighting (p<0.01); and at the lower comparison age (Figure 5B) for total visual reweighting (p<0.05) and intra-modal visual reweighting (p<0.05). Identical reweighting patterns for Head to Vdrive were observed for each group and for group differences (Figure 6 A, B).

#### Gains to the Tdrive

Even though children with DCD showed a marked developmental delay in visual reweighting compared to their TD peers, their ability to reweight to the Tdrive was comparable to their TD peers. For TD children, CoM to touch gain showed evidence of total (p<0.0001) and intra-modal (p<0.0001) reweighting at both upper comparison age of 10.8 years (Figure 5C, filled triangles) and lower comparison age of 6.6 years (Figure 5D, open face triangles; p<0.0001 for total and p<0.01 for intra- reweighting). Similarly for children with DCD, CoM to touch gain showed evidence of total reweighting (p<0.05) and intra-modal reweighting (p<0.01) at both the upper comparison age of 10.8 years (Figure 5C, filled squares) and lower comparison age of 6.6 years (Figure 5D, open face squares). The similar touch reweighting patterns between the two groups of children was supported by a lack of Group by Condition interactions for their touch reweighting. Similar Head to Tdrive reweighting patterns and lack of group differences were also observed (Figure 6C, D) with one exception that children with DCD at the lower comparison age of 6.6 years showed an atypical touch reweighting when touch amplitude was held constant while visual amplitude was changing (i.e., from T_2_V_2_ to T_2_V_8_).

### Fitted Phases at Comparison Ages

#### Phases to the Vdrive

An overall model test showed a significant main group effect (p<0.0001) for both CoM phase (Figure 7A, B; main group effect labeled above subplot A) and Head phase to the Vdrive (Figure 8A, B; main group effect labeled above subplot A). The phase responses of children with DCD to the Vdrive consistently lagged behind those of TD children at both comparison ages. Generally, CoM phase to the Vdrive showed no condition dependency for both TD children and children with DCD at both comparison ages (Figure 7A, B). For Head phase to Vdrive, there was no condition dependency for children with DCD (Figure 8A, B, square markers); while phases differed for TD children at the lower comparison age (Figure 8B, open face triangles) when visual amplitude was held constant while the touch amplitude changed (i.e., from T_8_V_2_ to T_2_V_2_) and when the visual scene and touch bar motion were at their highest or lowest amplitude (i.e., from T_8_V_2_ to T_2_V_8_). However, there was no Group by Condition interaction at the lower comparison age.

#### Phases to the Tdrive

Similar to phase responses to the Vdrive, the main group effect was significant (p<0.0001) for both CoM phase (Figure 7C, D; main group effect labeled above subplot C) and Head phase to the Tdrive (Figure 8C, D; main group effect labeled above subplot C). Similar to responses to the Vdrive, the phase responses of children with DCD to the Tdrive consistently lagged behind those of TD children at both comparison ages.

Generally, the phase response to the Tdrive was significantly different across conditions when reweighting to the Tdrive was observed (i.e., compare Figure 7C, D to Figure 5C, D; and compare Figure 8C, D to Figure 6C, D). Children with DCD showed some exceptions at the upper comparison age for the CoM phase (Figure 7C, filled squares) and for the Head phase (Figure 8C, filled squares). The Group by Condition interaction was also significant at the upper comparison age (Figure 7C, p<0.001 for total and p<0.10 for intra- and inter- reweighting; Figure 8C, p<0.01 to total and p<0.05 for intra-reweighting).

## Discussion

Multisensory integration has been implicated as a deficit in children with DCD. Here we provide the first quantitative evidence that indeed these children have a deficit in multisensory integration in a postural task with systematically varied touch and visual information. We provide three findings that implicate multisensory integration deficits in children with DCD. First, children with DCD show weak visual reweighting. At a young age (6.6 years), they reweight to touch but not vision. They only reweight to both touch and vision at an older age (10.8 years) while TD children reweight in both modalities as early as 4.2 years of age as previously reported [25]. Second, they do not show advanced multisensory fusion (i.e., inter-modal reweighting), even at 10.8 years of age, as previously observed in TD children [25]. Third, compared to TD children, children with DCD show a larger phase lag to both sensory modalities throughout the age range tested. Each finding is discussed below.

### Weak Visual Reweighting Signifies a Postural Control Deficit in Children with DCD

Only a general visual reweighting (i.e., total reweighting) was observed in older children with DCD at 10 years of age. This result is interpreted as a weak visual reweighting because both intra- and inter-modal reweighting were too small to be detected and reweighting was only observable between conditions in which stimulus amplitudes are most different (i.e., between T_8_V_2_ to T_2_V_8_). The difference in visual reweighting between TD children and children with DCD (i.e., weak visual reweighting in children with DCD) is not likely due to the greater triangle density of the visual display in the DCD experiment (see Methods). With a higher display density, one would expect a higher visual gain if other display parameters were kept the same (Lestienne, 1977, with checkerboard display) [52]. However, visual gain did not show an overall tendency to be higher for children with DCD (no main group effect). Thus, it is unlikely that visual display properties explain the weak reweighting in children with DCD. In contrast to visual reweighting, the touch reweighting pattern was similar between children with DCD and their TD peers. Both groups of children show adaptive touch reweighting across the age ranges tested that is mainly intra-modal. So why is visual reweighting more susceptible to a developmental disability such as DCD? We offer two possible explanations.

Our first explanation for this difference derives from a developmental perspective on the *postural body scheme* for upright stance control [53]. Postural body scheme, a representation of the body’s configuration and its relationships to the external world, requires the integration of multisensory information from sensors residing throughout the body. For example, proprioception conveys segment position information ranging from the head to the feet by stretch receptors, load receptors and sensors monitoring muscle effort, interacting bi-directionally (i.e., from head to feet, and from feet to head), and with other modalities such as vision and the vestibular system. The overall percept derived from multisensory fusion depends on which segment is used as the *reference frame* with respect to the external world. For infants who cannot stand independently, they are postulated to use the head as the reference frame and their postural response to moving visual information can be detected early in development [53]–[54]. After children acquire independent upright stance, it is proposed that the children use the supporting surface as a reference frame and proprioception conveys somatosensory information in an ascending fashion to integrate with other senses [54]. This claim is consistent with the findings that TD children [55] and children with DCD [20] rely more heavily on somatosensory cues for balance and postural sway increases in conditions with altered somatosensory information in the Sensory Organization Test [55]. Similarly, we previously reported a robust use of somatosensation (i.e., light touch), but not vision, for postural control in TD children [5], arguing, as did Riley and colleagues [56], that touch information at the fingertip provides body orientation information (an important component of the postural body scheme [53]). There is evidence that after about 6 years of age, children re-develop the ability to use the head, along with the visual/vestibular systems, as a reference frame during upright stance [54]. Our findings of comparable touch reweighting between TD children and children with DCD may reflect the importance of responding adaptively (e.g., reweighting) to a modality (i.e., somatosensation) that is used as a reference frame early on in childhood. Differences between visual reweighting between these two groups of children may be due to the longer developmental trajectory of using vision, compared to somatosensation, as a reference frame.

We also provide a second explanation for the weak visual reweighting from a developmental perspective of the role of the dorsal visual stream motion perception. Braddick and colleagues proposed that the dorsal stream (for motion perception) is more vulnerable than the ventral stream (for form perception) during development [57]. Deficits in visual motion perception have been reported in many developmental disabilities and especially established in children with dyslexia and with Fragile X syndrome [58]. For children with dyspraxia (poor motor planning), a diagnosis sometimes used interchangeably with DCD [59]–[60], conflicting results have been reported [58], [61]–[62]. While Sigmundsson’s group reported reduced sensitivity to dynamic random dot kinematogram [61]–[62], O’Brien and colleagues did not find reduced dorsal stream sensitivity [62]. This disagreement may stem from differences in how visual motion was presented [58]. Similar disagreement exists between ours and Wann’s finding [29] on the visual reweighting in children with DCD. Although we identify a weak visual reweighting as a signature postural control deficit, Wann and colleagues found that children with DCD show similar visual reweighting compared to TD children [29]. Three experimental design differences may contribute to the conflicting findings. First, Wann and colleagues used much larger amplitudes (approximately ±4.4, 8.8 and 13.2 cm) that may be easier to distinguish from self-motion, resulting in a down weighting of visual information. Second, they used visual movement in the anterio-posterior direction which is perceived earlier in development [63] than the medio-lateral visual translation movement we used. Third and most importantly, they only manipulated vision amplitude alone while we simultaneously manipulated touch and vision amplitudes. With the coexistence of a more robust reference frame, that is, touch, the reweighting to vision may be different. Our protocol may be more sensitive in identifying visual reweighting deficits in children with DCD and our finding supports Braddick’s view of dorsal stream vulnerability [57].

Although dorsal stream deficit may be a plausible explanation for the weak visual reweighting in children with DCD, it is not clear at what stage of visual processing the deficit occurs. A late stage involvement (i.e., higher visual cortex areas of motion processing) can be confirmed if the global motion processing deficit occurs without a deficit in early visual processing [58]. Nevertheless, indirect evidence of late stage cortical processing deficits for quiet stance control may be suggested from studies using fMRI during visuomotor activities in children with DCD [64]–[65]. Both Kashiwagi’s and Zwicker’s studies show different activation levels of higher visual cortex areas compared to TD children, and Zwicker et al. [64] interpret observed higher cortical activation pattern as greater reliance on vision information to complete motor tasks. Similarly, over reliance on vision information has been observed behaviorally for postural control in children with DCD [4]–[5]. Recent human brain imaging studies have established that these higher order cortical areas are also involved in the processing of motion information from other modalities such as somatosensation [66]–[68]. Based on the literature reviewed here, a multisensory integration deficit at a higher cortical level is a plausible explanation for our current findings.

### Multisensory Fusion Deficit Underlies the Compromised Postural Control in Children with DCD

Children with DCD do not show inter-modal reweighting, a more advanced form of multisensory integration (i.e., fusion) observed in older TD children at 10 years of age [25]. Inter-modal vision reweighting illustrates that the postural response to visual amplitude is also dependent on touch amplitude. This ability is interpreted as fusion of the two sensory modalities. What are the processes involved in multisensory fusion that may be impaired in children with DCD? We provide two possible mechanisms.

The first possible mechanism may involve the development of a feedback process that promotes bi-directional interactions between unimodal and polymodal cortical areas. Traditionally, sensory processing is considered to be a feedforward mechanism with information flow from the unimodal to the polymodal area [69]. New evidence indicates that feedback is possible from the higher visual cortex area (i.e., posterior parietal cortex) to middle temporal visual area [70]. This feedback process bears functional significance in that it influences immediate multisensory interaction [69] as well as bi-directional adaptation [71] (i.e., A modality affects B modality and vice versa). One example of an immediate multisensory interaction is that a moving visual distractor can modify the speed discrimination of simultaneously presenting tactile movement [68]. An example of multisensory adaptation is illustrated by a visual motion aftereffect that transfers to the touch modality, resulting in an illusionary movement of a stationary touch [71]. Similarly, a touch motion aftereffect also transfers to the visual modality highlighting the principle of bi-directional adaptation [71]. Deficits in multisensory interactions have been observed in children with clumsiness, a term sometimes used interchangeably with DCD, whereby the children show much worse length judgment under a cross-modal context than based on visual ot kinaesthetic information alone [72]. It has also been shown that clumsy children have problems transferring shape information between the haptic and visual modalities [73]. These studies of clumsy children, although not specific to the motion perception issue, support the notion that bi-directional multisensory integration may be impaired in children with DCD.

The second possible mechanism may involve the development of an adult-like computational efficiency for multisensory fusion. One example of a plausible computational model is Bayesian statistics that combine multisensory information in a statistically optimal fashion [36], [74]–[75]. In the Bayesian framework, a *prior* estimate is used as the basis for future estimation. It is argued that the *prior* estimate is not innate but learned. This argument is supported by studies in children showing that multisensory fusion takes a long time to develop [32], [34]–[35]. For example, 8-year-old children do not integrate vision and haptic information optimally for perceptual judgment. Instead, they use haptic information to judge size and vision to judge orientation [32]. Similarly, children younger than 8 years do not optimally integrate multisensory cues for navigation [35]. We also found that younger children of 4 years do not show multisensory fusion for quiet stance control [25]. Only at mid-childhood (i.e., 10 years old), an age very close to what has been reported from previous studies [32], [34]–[35] do children show multisensory fusion as indicated by inter-modal reweighting. However in children with DCD, no evidence of inter-modal reweighting exists to indicate multisensory fusion.

What might be the computational deficits underlying the non-optimal reweighting in children with DCD for quiet stance control? Our conceptualization of postural control is that of a feedback process with control based on the continuous estimation of body dynamics [27], [31], [40]. A steady state Kalman filter, another computational model that does not require a *prior* estimate, has been implemented successfully to model body dynamics of quiet stance [27], [31], [40]. We argue that the implementation of a Kalman filter for postural control may also need a relative long developmental trajectory to improve its computational proficiency, explaining the observation of multisensory fusion only after mid-childhood. Based on the framework of continuous state updating and feedback control, noise poses significant challenges to maintaining upright postural control which is intrinsically unstable. For children with DCD, variability is the cardinal feature prevalent in most motor tasks [7], [76]–[77]. The widely observed variability suggests a noisy motor control system that may interfere with optimal multisensory fusion.

### Group Difference in Phase Responses

For both visual-scene and touch-bar motion, phase responses of children with DCD lagged behind those of TD children. A similar DCD-vs-TD phase lag has previously been reported for responses to a visual moving room at a frequency of 0.17 Hz [29]. The Wann study and our study differed in both moving room direction (anterio-posterior in the Wann study, medio-lateral in our study) and the amount of visual re-weighting (similar for children with DCD and TD children in the Wann study, weaker for children with DCD in our study), suggesting that a DCD-vs-TD phase lag may be a robust property of responses to a moving visual scene. Also, our finding that a DCD-vs-TD phase lag is not specific to a single sensory modality suggests a general motor control deficit in children with DCD.

From a control theory perspective, because postural control is in a closed feedback loop, a DCD-vs-TD phase lag could potentially be due to differences in any component of the postural control system including sensory processing (sensor dynamic properties such as bandwidth or time delay), neural conduction delays, musculotendon dynamics, sensory integration, feedback control parameters, and nonlinear processes such as adaptation to predictable environmental motion. The data in the present study by themselves are not well suited to distinguish between these possibilities, since we measured gain and phase responses for each modality at a single frequency. In general, estimation of time delay based on frequency response properties requires gain and phase responses across a range of frequencies and a model of postural control (e.g., [22].

In the literature, although many studies have demonstrated timing deficits [11]–[12], sensory processing deficits [13]–[15], as well as multisensory integration deficits [3]–[5], [14]–[15], [20] in children with DCD, it is difficult to directly apply these behavioral or clinical findings to interpret the DCD-vs-TD phase lag observed in our current study. Generally speaking, little is known about how the individual components of the postural control feedback loop differ between children with DCD and TD children. For example, only very limited evidence exists for an increased somatosensory conduction delay in children with DCD; a small case study of two boys with DCD (ages 5 and 16 years) demonstrated a slower conduction of somatosensory evoked potentials [78]. For vision, a clinical study in 5- to 7-year-old children with DCD and TD children did not show group differences in visual evoked potential (VEP) delays to binocular high contrast grating stimuli [79]. However, this study did not test whether dorsal stream deficits (i.e., motion perception deficits) are present in children with DCD, and thus did not address the controversy that surrounds this question (see discussion above). In future studies, it would be valuable to quantify motion onset VEPs to investigate whether differences in visual motion processing contribute to the DCD-vs-TD phase lag. It would also be helpful to combine experiments that quantify the dynamics of other components of postural control with modeling to provide a more mechanistic explanation of the DCD-vs-TD phase lag.

### Conclusions

Multisensory reweighting is a critical adaptive ability for an individual to maintain quiet standing when sensory conditions change. With a recently developed protocol, we present simultaneous sinusoidal visual scene and touch bar movements at different frequencies and with differing amplitudes to simultaneously quantify sensory weights to vision and touch information in children with DCD compared to their TD peers. Our results reveal that children with DCD reweight to both touch and vision only at about 10.8 years of age while TD children reweight to both modalities as early as 4.2 years of age. Children with DCD demonstrate a general and weak visual reweighting which can be explained from two developmental perspectives: one being the development of *postural body scheme*; and the other being the dorsal stream deficit in children with developmental disabilities. Our results also reveal that children with DCD do not show advanced multisensory fusion (i.e., inter-modal reweighting). Our findings support the view that multisensory integration (i.e., fusion) is slow to develop and not optimal until mid-childhood. In children with DCD whose neural processing for multisensory integration is impaired, the process of achieving optimal adult-like neural computation to estimate postural orientation is markedly delayed compared to their TD peers. On top of the multisensory fusion deficit, these children also show significant phase lag responses in their postural response to sensory stimuli.
